# Post-thrombotic syndrome after thrombotic venous thoracic outlet syndrome

**DOI:** 10.1016/j.jvsv.2026.102639

**Published:** 2026-10-09

**Authors:** Samy Benhalima, Giovanni Gautier, Frédéric Douane, Alban Fouasson-Chailloux, Guillaume Gadbled, Benjamin Espinasse, Blandine Maurel, Marie Morard, Olivier Espitia

**Affiliations:** a Department of Internal and Vascular Medicine, Nantes Université, CHU Nantes, Nantes, France; b Department of Vascular Medicine, European Hospital Georges Pompidou, Paris, France; c Department of Radiology Medicine, Nantes Université, CHU Nantes, Nantes, France; d Department of Physical Medicine and Rehabilitation, Nantes Université, CHU Nantes, Nantes, France; e Department of Orthopaedics, Nantes Université, CHU Nantes, Nantes, France; f Department of Vascular Medicine, Brest Université, CHU, Brest, France; g Brest Université, Inserm, UMR 1304, GETBO, Brest, France; h Department of Vascular Surgery, Nantes Université, CHU Nantes, Nantes, France; i Nantes Université, CHU Nantes, CNRS UMR 6291, INSERM UMR 1087, l'institut du thorax Team III Vascular and Pulmonary Diseases, Nantes, France

**Keywords:** Thoracic outlet syndrome, Post-thrombotic syndrome, Upper extremity vein thrombosis, Subclavian vein thrombosis, Axillary vein thrombosis

## Abstract

**Background:**

Thrombotic venous thoracic outlet syndrome (T-vTOS), including Paget-Schrotter syndrome, is a rare form of upper extremity deep vein thrombosis that mainly affects young, active individuals. Its optimal management remains debated, and the medium-term outcomes have been poorly documented.

**Methods:**

This retrospective study included consecutive patients diagnosed with T-vTOS at the vascular medicine department of a university hospital between 2014 and 2024. Patients were stratified according to the presence of post-thrombotic syndrome (PTS) using the modified Villalta score at the last follow-up (<5 vs ≥5). Clinical characteristics, therapeutic strategies, and outcomes were systematically assessed through a standardized evaluation performed by the same physician. Univariable and multivariable logistic regression analyses, using Firth’s penalized-likelihood method to account for the limited number of events, were performed to identify the factors associated with PTS.

**Results:**

A total of 50 patients were included: 28 with a Villalta score of <5 and 22 with a Villalta score of ≥5 at the last follow-up. Patients with a Villalta score of <5 achieved higher rates of complete subclavian vein recanalization at follow-up (63% vs 32%; *P* = .045) and were less likely to have persistent subclavian vein occlusion (26% vs 59%; *P* = .023). Patients with a Villalta score of ≥5 more often required prolonged anticoagulation (73% vs 29%; *P* = .004) and more frequently underwent acute-phase endovascular treatment (36% vs 4%; *P* = .007). In the multivariable analysis, endovascular treatment alone without thoracic outlet surgery was associated with an increased risk of PTS (odds ratio, 10.6; 95% confidence interval, 1.15, 98.09) (*P* = .037), whereas no association was found between the risk of PTS and patients who had undergone both endovascular treatment and TOS surgery (odds ratio 1.3; 95% confidence interval, 0.26, 6.87) (*P* = .736).

**Conclusions:**

This study highlights the central role of venous patency in the clinical course of T-vTOS, with persistent subclavian vein occlusion associated with an increased risk of PTS. The association observed between acute-phase endovascular treatment alone and the risk of PTS does not support a clear benefit of this strategy. However, endovascular therapy in conjunction with surgical decompression of the thoracic outlet may reduce the risk of PTS.

**Article Highlights**

• **Type of Research:** Single-center retrospective cohort study
• **Key Findings:** Endovascular intervention alone, without surgical decompression of the thoracic outlet, was associated with an increased risk of post-thrombotic syndrome (odds ratio, 10.6; *P* = .037).
• **Take Home Message:** An integrated approach involving endovascular intervention plus thoracic outlet syndrome surgery is favorable.

Primary upper extremity deep vein thrombosis (DVT) is an uncommon disease with an incidence of 1 to 2 per 100,000 people.^1^, ^2^ Among the upper extremity DVT conditions, Paget-Schroetter syndrome (PSS), a form of venous thoracic outlet syndrome (vTOS), is characterized by the effort-induced thrombosis of the axillary-subclavian veins due to anatomical compression at the costoclavicular junction; it predominantly affects young, active individuals, including athletes and manual laborers.^3^, ^4^ While the acute phase is managed with anticoagulation (AC) and endovascular thrombolysis, the long-term risk of post-thrombotic syndrome (PTS) remains a significant concern.^5^, ^6^, ^7^, ^8^ Despite its clinical relevance, the optimal management strategy to prevent PTS in PSS remains poorly defined, with persistent uncertainties regarding the timing of surgical decompression, the role of endovascular interventions, and the optimal medical regimen.

The current treatment paradigms for PSS typically involve catheter-directed thrombolysis (CDT), followed by first-rib resection (FRR) with or without venoplasty; this strategy is supported by observational data demonstrating superior symptom resolution and patency rates compared with AC alone. A systematic review and meta-analysis reported that 96% of patients treated with CDT + FRR were symptom-free at the last follow-up compared with only 54% treated with AC alone.^9^ However, these findings were based on low-certainty evidence, with considerable heterogeneity and a lack of randomized controlled trials, indicating the absence of high-quality comparative data to guide clinical decision-making.^9^

The surgical approach for thoracic outlet decompression also remains debated. A meta-analysis comparing infraclavicular, supraclavicular, paraclavicular, and transaxillary approaches found that infraclavicular, supraclavicular, and paraclavicular techniques achieved higher primary patency rates (95%-100%) and clinical improvement (84%-100%) than the transaxillary method (patency rate of 69%, improvement of 88%).^10^ Yet, even within these approaches, variability in outcomes persists, which is likely due to differences in patient selection, timing of surgery relative to thrombolysis, and adjunctive endovascular techniques.^10^, ^11^ Furthermore, no consensus exists on the optimal timing of FRR after CDT; some centers advocate for early decompression (within 30 days) to reduce rethrombosis risk, whereas other centers advocate delaying surgery by 6 to 12 weeks to allow venous remodeling.^12^, ^13^

The role of nonsurgical management in PSS is equally contentious. A cohort study demonstrated that 94% of patients treated with CDT + AC remained free of PTS at the mean follow-up of 109 months, with 100% venous patency on ultrasound images and 78% completely asymptomatic.^14^ These results challenge the dogma that surgical decompression is mandatory for all patients with PSS, suggesting that a subset of patients may achieve durable symptom relief with endovascular and medical therapy alone.^14^, ^15^ However, the study was limited by its small sample size and the lack of a control group, with the question of whether long-term AC, thrombolysis technique, or patient-specific factors influence outcomes remaining unanswered.^14^

From an endovascular perspective, emerging data suggest that single-session thrombectomy devices offer higher primary patency and lower reintervention rates compared with CDT, with symptom resolution in up to 85% of patients at 6 months.^16^ Yet, these findings are exploratory, derived from retrospective single-center cohort studies with short follow-up, and do not address the long-term risk of PTS or the need for subsequent surgical decompression.^16^

Medical management lacks standardization. While 3 to 6 months of AC is commonly prescribed post FRR,^4^ some centers extend the therapy indefinitely in high-risk patients.^14^, ^17^ The optimal antithrombotic regimen and the role of physical therapy in preventing PTS remain unclear.^18^

PTS is among the most frequent long-term complications; the reported frequencies vary widely across studies, from approximately 6% to 15% in pooled analyses combining primary and secondary upper extremity DVT^5^, ^19^ to up to 60% in series restricted to primary disease.^20^ This variability partly reflects the historical absence of a validated diagnostic score for upper extremity PTS,^8^ indicating the need for obtaining data specifically on thrombotic venous thoracic outlet syndrome (T-vTOS); in T-vTOS, a persistent compressive mechanism may carry a distinct risk profile compared with catheter-related or other secondary thrombosis. The 2026 American Venous Forum clinical practice guideline on the care of patients with upper extremity DVT recommends that VTOS-associated upper extremity DVT be managed with FRR.^21^ While CDT + FRR appears to yield the best functional outcomes in PSS, the optimal surgical approach, timing of intervention, necessity of surgery in all patients, and long-term medical management remain unresolved.

This study compared management, venous patency, and clinical manifestations stratified according to the presence of PTS after T-vTOS.

## Methods

### Study design and setting

We conducted a retrospective study at the vascular medicine department of a university hospital, including all consecutive patients diagnosed with subclavian vein thrombosis due to vTOS between January 2014 and December 2024. Patients were eligible if TOS was diagnosed either at the time of thrombosis or after venous recanalization.

PSS/effort thrombosis was defined as subclavian vein thrombosis without favoring factors apart from repeated arm movements. Patient with upper extremity DVT and vTOS were also included, even if no physical activity involving the upper limbs had been identified during the medical interview just before vein thrombosis onset.

The exclusion criteria were as follows: upper extremity DVT associated with the presence of a central line or a PICC line; venous thrombosis associated with the presence of pacemaker leads; and malignancy and upper extremity DVT following surgery or trauma to the upper limb.

### Clinical evaluation

All patients were assessed in a dedicated outpatient TOS consultation conducted by the same physician (G.G.), ensuring the standardized evaluation and follow-up. The diagnosis was established based on a combination of clinical history and a duplex ultrasound (DUS) showing venous thrombosis reaching at least the subclavian vein, with the head of the thrombus below the costoclavicular junction (Fig). The typical historical features included repeated upper limb movements predisposing to Paget-Schroetter syndrome or symptom onset upon awakening.

**Fig:**
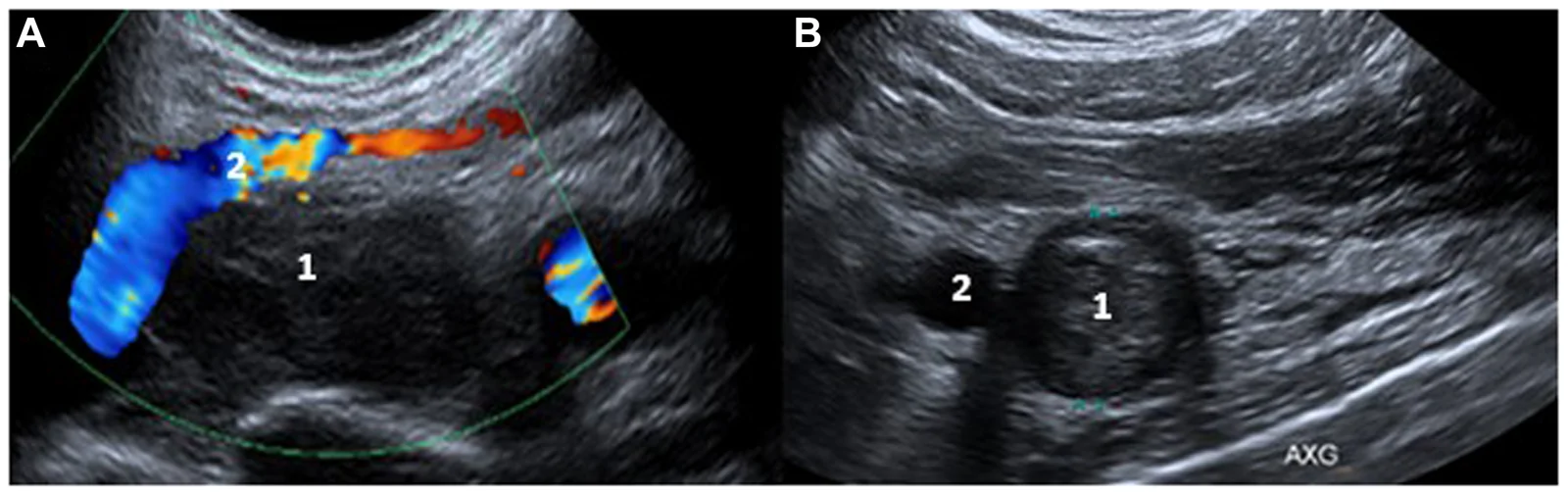
Acute vein thrombosis associated with venous thoracic outlet syndrome. **(A)** Longitudinal image with the probe above the clavicle: (*1*) Occlusive thrombosis of the subclavian vein and (*2*) development of collateralization from the external jugular vein. **(B)** Transverse ultrasound image with the probe below the clavicle: (*1*) Occlusive thrombosis of the axillary vein and (*2*) Axillary artery free of lesions.

Each patient underwent venous imaging assessment with at least a DUS with an evaluation of the thrombus head beneath the costoclavicular space on DUS, of venous stenosis under the costoclavicular junction on end-of-treatment venography, or dynamic compression of the subclavian vein during arm abduction or head rotation on DUS after recanalization. Clinical assessments with the modified Villalta score for upper limbs were repeated in each follow-up visit.

Acute-phase endovascular treatment consisted of pharmaco-mechanical thrombectomy or mechanical thrombectomy, the choice depending on the delay since diagnosis (<14 days for pharmaco-mechanical thrombectomy). Chronic endovascular treatment consisted of mechanical recanalization through balloon angioplasty.

At the last follow-up, patients with a Villalta score of <5 were included in the no PTS (No-PTS) group, and patients with a Villalta score of ≥5 were included in the PTS group.^19^

### Imaging investigations

Other than DUS, some patients underwent additional imaging: cervical spine radiography for detecting cervical rib, enlarged transverse process, or rib synostosis and computed tomography venography or magnetic resonance imaging that were performed selectively depending on the clinical context.

### Outcomes

This study focused on (1) clinical and imaging characteristics at the T-vTOS diagnosis, (2) the therapeutic strategies employed (AC, thrombectomy, surgical decompression, or combined approaches), and (3) clinical and imaging outcomes during follow-up.

### Ethical considerations

This study was conducted in compliance with the Declaration of Helsinki principles and received ethics approval from Groupe Nantais d’Ethique et de Soins (GNEDS), the local ethics committee of the University Hospital of Nantes (2024-0301). Each patient included in this study received written information, and no patient objected to this study. The need for written informed consent was waived by the ethics committee because of the retrospective study design (French public health code article: L 1121-1).

### Statistical analysis

Descriptive statistics were used to summarize the baseline clinical and paraclinical characteristics. Continuous variables are expressed as median and interquartile range (Q1-Q3: 25-75) after the assessment of distribution; categorical variables are expressed as counts and percentages. Comparisons between the two PTS groups, defined by a modified Villalta score of <5 (No PTS) vs ≥5 (PTS) at the last follow-up, were performed using the Fisher exact test for categorical variables and the Welch two-sample *t*-test or the Mann-Whitney *U* test for continuous variables, according to the data distribution.

To identify the factors associated with PTS, multivariable logistic regression analyses were performed. Given the limited number of PTS events (n = 22) relative to the number of candidate variables, two multivariable models were constructed (using Firth’s penalized-likelihood method to account for the limited number of events) rather than developing a single comprehensive model, in order to limit overfitting: a baseline model including the anatomical and demographic variables available at diagnosis and a management-related exploratory model including acute-phase endovascular treatment and the combination of endovascular treatment and surgical treatment. Logistic regression models were used to assess the variables associated with PTS in the multivariable analysis. Odds ratios (ORs) are reported with 95% confidence intervals (CIs).

All statistical tests were two-sided, and a *P* value of <.05 was considered statistically significant. Descriptive statistics and univariable comparisons were performed using the BiostatTGV online platform (https://biostatgv.sentiweb.fr/); logistic regression analyses were performed using R software.

## Results

A total of 50 patients with T-vTOS were included: 28 (56%) without PTS and 22 (44%) with PTS at the last follow-up. The baseline characteristics are presented in Table I. The median age was 31.0 years (24.0-43.5) in the No-PTS group and 39.5 years (29.2-47.5) in the PTS group (*P* = .256). The sex distribution was similar between the groups (*P* = .393). Half of the patients in the No-PTS group were athletes compared with 27% in the PTS group (*P* = .148). Manual occupations were similarly represented in both groups (29% vs 32%; *P* > .99). No patient had a personal history of venous thromboembolism. Thrombophilia was identified in 18% of patients without PTS and 9% of those with PTS (*P* = .444). Oral contraception at the time of thrombosis was reported in 44% of women without PTS and 33% of those with PTS (*P* = .691). Pulmonary embolism was observed at diagnosis in 11% and 14% of patients in the No-PTS and PTS groups, respectively (*P* > .99).

**Table I:** Baseline characteristics according to post-thrombotic syndrome status

Characteristics	T vTOS (n = 50)	No PTS (n = 28)	PTS (n = 22)	*P* value
Age, years, median (Q1-Q3)	35.0 (24.3-43.8)	31.0 (24.0-43.5)	39.5 (29.2-47.5)	.256
Men, n/N (%)	25/50 (50%)	12/28 (43%)	13/22 (59%)	.393
Athletes, n/N (%)	20/50 (40%)	14/28 (50%)	6/22 (27%)	.148
Manual workers, n/N (%)	15/50 (30%)	8/28 (29%)	7/22 (32%)	>.99
Thrombophilia, n/N (%)	7/50 (14%)	5/28 (18%)	2/22 (9%)	.444
Oral contraception at DVT, n/N (%)	10/25 (40%)	7/16 (44%)	3/9 (33%)	.691
Pulmonary embolism at diagnosis, n/N (%)	6/50 (12%)	3/28 (11%)	3/22 (14%)	>.99
Distal extent of thrombosis at diagnosis				
Subclavian, n/N (%)	12/49 (24%)	9/27 (33%)	3/22 (14%)	.182
Axillary, n/N (%)	21/49 (43%)	9/27 (33%)	12/22 (55%)	.158
Humeral, n/N (%)	10/49 (20%)	7/27 (26%)	3/22 (14%)	.478
Basilic and/or cephalic, n/N (%)	6/49 (12%)	2/27 (7%)	4/22 (18%)	.388
Cervical spine X ray, n/N (%)	30/50 (60%)	17/28 (61%)	13/22 (59%)	>.99
Enlarged transverse process, n/N (%)	7/30 (23%)	4/17 (24%)	3/13 (23%)	>.99
Cervical rib, n/N (%)	2/30	1/17	1/13	>.99

*DVT*, Deep vein thrombosis; *PTS*, post-thrombotic syndrome; *T vTOS*, thrombotic venous thoracic outlet syndrome.

Table II presents the therapeutic management. Regarding AC, the median treatment duration was 14.5 months (6.7-35.6) in the No-PTS group and 23.2 months (13.1-36.6) in the PTS group, with no significant difference between the groups (*P* = .14). At the last follow-up, patients with PTS were more frequently still receiving AC than those without PTS (73% vs 29%; *P* = .004). The type of initial anticoagulant (low-molecular-weight heparin, vitamin K antagonist, or direct oral anticoagulant) did not differ significantly between the groups.

**Table II:** Management according to post-thrombotic syndrome status

Management	T vTOS (n = 50)	No PTS (n = 28)	PTS (n = 22)	*P* value
First-rib resection, n/N (%)	13/50 (26%)	9/28 (32%)	4/22 (18%)	>.99
+ Subclavius tenotomy, n/N (%)	5/13	4/9	1/4	>.99
+ Pectoralis minor tenotomy, n/N (%)	1/13	0/9	1/4	>.99
+ Anterior scalene tenotomy, n/N (%)	9/13	6/9	3/4	.339
Delay between diagnosis and surgery in months, median (Q1-Q3)	15.8 (8.2-22.8)	20.5 (10.6-36.0)	15.6 (12.4-18.0)	>.99
Acute-phase endovascular treatment, n/N (%)	9/50 (18%)	1/28 (4%)	8/22 (36%)	.007
Delay from diagnosis in days, median (Q1-Q3)	13.0 (10.0-13.0)	13.0 (-)	11.5 (10.0-13.0)	.99
Chronic endovascular treatment, n/N (%)	6/50 (12%)	4/28 (14%)	2/22 (9%)	.683
Initial anticoagulation, n/N (%)	50/50 (100%)	28/28 (100%)	22/22 (100%)	>.99
LMWH	7/50 (14%)	3/28 (11%)	4/22 (18%)	.684
VKA	2/50 (4%)	1/28 (4%)	1/22 (5%)	>.99
DOAC	35/50 (70%)	18/28 (64%)	17/22 (77%)	.367
Apixaban	11/35 (31%)	7/18 (39%)	4/17 (24%)	.471
Rivaroxaban	24/35 (69%)	11/18 (61%)	13/17 (76%)	.471
Duration of anticoagulation in months, median (Q1-Q3)	16.2 (7.8-35.9)	14.5 (6.7-35.6)	23.2 (13.1-36.6)	.139
Patients still on anticoagulation at the last follow-up, n/N (%)	24/50 (48%)	8/28 (29%)	16/22 (73%)	.004

*DOAC*, Direct oral anticoagulant; *LMWH*, low-molecular-weight heparin; *PTS*, post-thrombotic syndrome; *T vTOS*, thrombotic venous thoracic outlet syndrome; *VKA*, vitamin K antagonist.

Chronic endovascular treatment was performed in two patients with PTS and four patients without PTS (*P* = .683). In the No-PTS group, nine patients (32%) underwent FRR compared with 4 (18%) in the PTS group (*P* = .339). Acute-phase endovascular treatment was more frequently performed in the PTS group: eight patients (36%) vs 1 (4%) in the No-PTS group (*P* = .007).

Table III presents venous patency at the last follow-up. The follow-up duration was similar between the two groups: a median duration of 45.0 months (22.0-61.5) for patients without PTS vs 29.0 months (15.0-48.0) for patients with PTS (*P* = .183). Patients in the PTS group had significantly more persistent subclavian vein occlusion at the last Doppler ultrasound scan than those in the No-PTS group (59% vs 26%; *P* = .023), and patients with PTS had significantly less complete recanalization than patients without PTS (32% vs 63%; *P* = .045). For axillary vein involvement, occlusion at the last Doppler ultrasound scan was more frequent in the PTS group (26% vs 5%; *P* = .18). During follow-up, venous thrombosis recurrence occurred in four patients without PTS (14%) and five patients with PTS (23%) (*P* = .481); all recurrences were ipsilateral in both groups. AC had been discontinued before recurrence in three of four patients (75%) in the No-PTS group and one of five patients (20%) in the PTS group (*P* = .206). The median interval between AC discontinuation and recurrence was 5.0 months in the No-PTS group and 16.0 months in the PTS group.

**Table III:** Follow-up according to post-thrombotic syndrome status

Follow-up	T vTOS (n = 50)	No PTS (n = 28)	PTS (n = 22)	*P* value
Follow-up duration in months, median (Q1-Q3)	36.0 (19.0-55.8)	45.0 (22.0-61.5)	29.0 (15.0-48.0)	.183
Delay between diagnosis and last Doppler ultrasound scan in months, median (Q1-Q3)	31.0 (13.0-48.0)	32.0 (13.8-50.5)	29.0 (13.0-45.0)	.731
Subclavian vein thrombosis at diagnosis, n/N (%)	49/50 (98%)	27/28 (96%)	22/22 (100%)	>.99
Occluded at the last Doppler ultrasound scan, n/N (%)	20/49 (41%)	7/27 (26%)	13/22 (59%)	.023
Partial recanalization at the last Doppler ultrasound scan, n/N (%)	5/49 (10%)	3/27 (11%)	2/22 (9%)	>.99
Complete recanalization at the last Doppler ultrasound scan, n/N (%)	24/49 (49%)	17/27 (63%)	7/22 (32%)	.045
Axillary vein thrombosis at diagnosis, n/N (%)	38/50 (76%)	19/28 (68%)	19/22 (86%)	.186
Occluded at the last Doppler ultrasound scan, n/N (%)	6/38 (16%)	1/19 (5%)	5/19 (26%)	.18
Partial recanalization at the last Doppler ultrasound scan, n/N (%)	18/38 (47%)	9/19 (47%)	9/19 (47%)	>.99
Complete recanalization at the last Doppler ultrasound scan, n/N (%)	14/38 (37%)	9/19 (47%)	5/19 (26%)	.313
Number of recurrences, n/N (%)	9/50 (18%)	4/28 (14%)	5/22 (23%)	.481
Ipsilateral, n/N (%)	9/9	4/4	5/5	>.99
Continuous venous flow through collateral circulation, n/N (%)	13/50 (26%)	5/28 (18%)	8/22 (36%)	.197

*PTS*, Post-thrombotic syndrome; *T vTOS*, thrombotic venous thoracic outlet syndrome.

At the last follow-up, the modified Villalta score was higher in the PTS group as expected, with a median score of 7.0 (6.0-9.0) vs 3.0 (0.0-4.0) in the No-PTS group.

In multivariate analysis, endovascular treatment alone without thoracic outlet surgery was associated with an increased risk of PTS (OR, 10.6; 95% CI, 1.15, 98.09) (*P* = .037), whereas no association was observed between PTS risk and patients who had undergone both endovascular treatment and TOS surgery (OR, 1.3; 95% CI, 0.26, 6.87) (*P* = .736).

## Discussion

This study cohort represents one of the larger T-vTOS cohorts, comprising 50 patients (in whom 22 had PTS) and benefiting from systematic clinical and vascular imaging follow-up over a 10-year period. Several methodological strengths should be emphasized. First, patients were evaluated in a dedicated vascular consultation by the same experienced physician, which minimized interobserver variability and ensured a standardized diagnostic and follow-up approach. In contrast, previous series have reported highly heterogeneous management strategies, reflecting the absence of standardized guidelines.^22^ After T-vTOS, follow-up was close and repeated, allowing for a reliable medium-term assessment of both venous patency and post-thrombotic sequelae. The use of the modified Villalta score provided a structured evaluation of PTS, pending the validation of a PTS-specific score for the upper limbs.^23^

The management of T-vTOS is not standardized, and the choice of interventional, surgical, or medical strategies in the acute or chronic setting primarily depends on the local practice. According to the current treatment guidelines on venous thrombosis and prevention, the preferred therapy for primary upper extremity DVT is conservative therapy with AC, whereas interventional therapies such as pharmacological or/and mechanical thrombectomy and surgery are only recommended in selected patients in an expert center.^24^, ^25^

Our findings highlight the central role of venous patency in the clinical course of T-vTOS. Persistent subclavian vein occlusion at the last follow-up was significantly more frequent in the PTS group, and complete recanalization was significantly less frequent in the PTS group than in the No-PTS group (32% vs 63%), confirming venous patency as the strongest correlate of long-term outcomes in our cohort, as also reported in the literature.^26^, ^27^, ^28^

The venous thrombosis burden may influence PTS, a more extensive thrombosis, extending to the veins of the arms, may have a different pathophysiology from that of thromboses confined to the axillary and subclavian veins. Various hypotheses can be proposed, such as a more severe obstructive mechanism or more chronic venous damage favoring greater extension. It may also be due to a late diagnosis of thrombosis, leading to a less effective response to anticoagulant treatment.

FRR was numerically more frequent in the No-PTS group, but this difference did not reach statistical significance (32% vs 18%). This finding should therefore be interpreted with caution; the small sample size and the retrospective nonrandomized allocation to surgery—mainly driven by clinical severity and physician judgement—limit any causal interpretation.

In contrast, patients with PTS had more frequent persistent occlusion, were more often maintained on long-term AC, and showed a higher prevalence of post-thrombotic sequelae. The longer duration of AC in the PTS group was linked to concerns about a highly symptomatic thrombotic recurrence in patients who had not developed a satisfactory collateral network and who presented with significant obstructive syndrome. Acute-phase endovascular treatment was significantly more frequent in the PTS group (36% vs 4%). This association should be interpreted with caution; it may partly reflect confounding by indication, as this strategy could have preferentially been offered to patients with a higher thrombus burden or a more severe presentation at diagnosis, but it may also suggest that acute-phase endovascular treatment does not confer the expected long-term benefit in this setting. This hypothesis-generating finding warrants confirmation in larger, prospective studies before any conclusion can be drawn on the actual efficacy of this strategy.

Similarly, ongoing AC at the last follow-up in the PTS group most likely reflects persistent venous occlusion and continued clinical concern rather than being a cause of poor outcomes. These observations are in line with previous reports describing the importance of decompression surgery in improving long-term patency and functional results, although our data alone cannot establish a causal benefit of surgery. They also describe the limitations of medical therapy alone, which is associated with a higher risk of chronic venous obstruction and disabling symptoms. In our study, DVT recurrence occurred on the same side as the initial thrombosis, highlighting the role of the underlying anatomical obstruction and residual venous lesions in the mechanisms of recurrence. Klejn al^12^ suggest that early thoracic outlet decompression within 2 weeks after thrombolysis for primary upper extremity DVT is as safe and effective as delayed surgery, while potentially reducing the AC time and health care costs.

The multivariable analysis showed that, in addition to AC, endovascular treatment without TOS surgery was associated with the onset of PTS. This finding is consistent with the pathophysiology of thrombosis, in which an anatomical obstruction is a major factor leading to increased venous resistance.

As described in a previous study,^22^ surgical treatment seems to be beneficial in T-vTOS.^29^ FRR remains a major surgical procedure, and the postoperative outcomes substantially depend on the surgical team’s experience, as highlighted by Sorber et al.^30^ Similarly, a 10-year surgical experience in PSS management has been reported.^31^

Several limitations must be acknowledged. The retrospective design inherently introduces information and selection bias for interventional management. Selection bias is a well-recognized issue in the literature on vTOS-associated upper extremity DVT.^32^ Furthermore, patient assessment was not conducted in a blinded manner with regard to the management strategies, which may have led to bias in the assessment. However, treatments were offered depending on the residual symptoms and the feasibility of intervention. Another limitation is the absence of a standardized validated scoring system for PTS in the upper extremity when assessing these patients. We used the modified Villalta score, originally developed for the lower limb, which may not fully capture the specificities of upper extremity involvement. Recently, an international Delphi study has proposed a score for upper extremity PTS that would enable the more effective assessment of PTS; it has yet to be validated.^23^ Given the small sample size, these results should be interpreted with caution, in particular with regard to surgical treatments.

Finally, the management strategies evolved over the study period, which may have influenced the outcomes. The physician performing the clinical assessment was also aware of each patient's treatment history, which may have introduced observer bias in the scoring of Villalta items with a subjective component.

Despite these limitations, our study provides meaningful insights into the medium-term outcomes of T-vTOS. It emphasizes the value of a standardized approach with systematic follow-up and objective clinical assessment to better characterize this rare but functionally disabling condition.

Given the paucity of high-quality comparative data, combined with the heterogeneity in treatment protocols and outcome reporting, prospective studies and randomized trials are warranted to establish evidence-based guidelines.

This case-series with the systematic and standardized follow-up of patients with T-vTOS confirmed venous patency as the strongest correlate of post-thrombotic outcome and suggests the association between early decompression surgery and improved outcomes. The association observed between endovascular treatment without TOS surgery and PTS risk does not support a clear benefit of this strategy. Despite the limitations inherent to a retrospective design and the rarity of the disease, these findings support an integrated medico-surgical approach. Further prospective studies should be conducted to refine the management strategies for T-vTOS.

## Author Contributions

Conception and design: SB, GiG, MM, OE

Analysis and interpretation: SB, GiG, FD, BE, OE

Data collection: SB, GiG, FD, AF, GuG, BE, BM, MM, OE

Writing the article: SB, GiG, MM, OE

Critical revision of the article: SB, GiG, FD, AF, GuG, BE, BM, MM, OE

Final approval of the article: SB, GiG, FD, AF, GuG, BE, BM, MM, OE

Statistical analysis: SB, OE

Obtained funding: Not applicable

Overall responsibility: OE

## Funding

None.

## Disclosures

None.
